# Bispectrum Features and Multilayer Perceptron Classifier to Enhance Seizure Prediction

**DOI:** 10.1038/s41598-018-33969-9

**Published:** 2018-10-19

**Authors:** Elie Bou Assi, Laura Gagliano, Sandy Rihana, Dang K. Nguyen, Mohamad Sawan

**Affiliations:** 1 Polystim Neurotech Lab, Institute of Biomedical Engineering, Polytechnique Montreal, Montreal, QC Canada; 2 Biomedical Engineering Department, Holy Spirit University of Kaslik (USEK), Jounieh, Lebanon; 3 University of Montreal Hospital Center (CHUM), University of Montreal, Montreal, QC Canada

## Abstract

The ability to accurately forecast seizures could significantly improve the quality of life of patients with drug-refractory epilepsy. Prediction capabilities rely on the adequate identification of seizure activity precursors from electroencephalography recordings. Although a long list of features has been proposed, none of these is able to independently characterize the brain states during transition to a seizure. This work assessed the feasibility of using the bispectrum, an advanced signal processing technique based on higher order statistics, as a precursor of seizure activity. Quantitative features were extracted from the bispectrum and passed through two statistical tests to check for significant differences between preictal and interictal recordings. Results showed statistically significant differences (p < 0.05) between preictal and interictal states using all bispectrum-extracted features. We used normalized bispectral entropy, normalized bispectral squared entropy, and mean of magnitude as inputs to a 5-layer multilayer perceptron classifier and achieved respective held-out test accuracies of 78.11%, 72.64%, and 73.26%.

## Introduction

Epilepsy is a chronic condition characterized by recurrent ‘unpredictable’ seizures. While the first line of treatment consists of long-term drug therapy, more than a third of patients are pharmacoresistant^1^. The availability of several new antiepileptic drugs over the last two decades helped in reducing the risk of adverse events but their impact on the rate of seizure control is only modest^2^. In addition, recourse to epilepsy surgery remains low in part due variable success rates depending on the complexity of the case at hand, accessibility, and persisting negative attitudes towards it and fear of complications^3,4^.

Predicting the possible occurrence of seizures is an unmet medical need and such capability can lead to novel therapeutic avenues to treat patients with refractory epilepsy. Unlike seizure detection, seizure prediction can foresee the possibility of future occurrence of seizure in advance, thus allowing medical intervention to potentially prevent the seizures or reduce their magnitude and/or frequency. However, the ability to accurately identify the pre-seizure state remains elusive. Despite several attempts to identify a specific and unique feature that can be used to predict seizures, no single characteristic has been established as a potential and universal precursor of epileptic seizure activity^5–7^.

The commonly used feature in seizure prediction, the spectral band power, is derived from the frequency domain characteristics of electroencephalography (EEG) signals^8^. It quantifies amplitude modulations across time, within the defined frequency bands. While the spectral band power displays phase changes, it cannot identify interactions among frequency components of the signal. However, information regarding multi-frequency behaviors can be captured by more complex metrics, related to the concept of cross-frequency coupling (CFC)^9^. Recently, Alvarado-Rojas *et al*. (2014) introduced a new measure of brain excitability based on phase-amplitude coupling (PAC), consisting of a slow (delta, theta) modulation of high (gamma) frequency intracranial EEG (iEEG) signal’s components^10^. They reported promising prospective results suggesting that preictal PAC modulations may be significant for the whole group of patients (p < 0.05). We later showed the existence of significant difference in mean PAC distribution between the preictal and interictal states on bilateral canine iEEG recordings^11^. Furthermore, Bandarabadi *et al*. (2015) reported promising results with a new bivariate feature (although not termed as CFC) quantifying the cross-power information between two different frequency bands (assessed in terms of power spectral density) and two different channels^12^. Overall, these findings suggest that seizure prediction may be possible using cross-frequency coupling. In contrast to the previously discussed measures, higher order spectral measures based on CFC have been proposed to be the carrier mechanism for the relationship between global and local neuronal processes^9^.

The bispectrum is an advanced signal processing technique based on higher order statistics which considers both the amplitude and the degree of phase coupling of a signal. In contrast to traditional power spectrum, which quantifies the power of a time series over frequency, higher order spectral (HOS) analysis employs the Fourier transform of higher order correlation functions to explore the existence of quadratic (and cubic) non-linear coupling information. Although the bispectrum has shown promising results within the context of seizure detection and EEG signals classification^13^, it has not yet been used for seizure prediction. In this work, we investigated the suitability of the bispectrum in quantifying changes between the interictal and preictal states. Adequate statistical tests were employed to assess if there are significant differences among the quantified changes. A seizure prediction algorithm employing a multilayer perceptron (MLP) neural network was used, showing good performances in classifying preictal and interictal samples. The seizure prediction algorithm was designed and tested to perform an automatic classification of preictal and interictal samples. Such algorithm could eventually be embedded in an advisory/intervention closed-loop system, resulting in a life-changing solution for patients with refractory epilepsy.

## Methods

All methods were carried out in accordance with relevant regulations and guidelines. The procedure for data acquisition and distribution had prior approval of the University of Minnesota Institutional Animal Care and Use Committee where the animals are maintained^14^.

### Database

HOS analysis features were extracted from interictal and preictal iEEG recordings of 3 mixed hounds implanted with the NeuroVista ambulatory monitoring device. These recordings were downloaded from the NIH-sponsored international electrophysiology portal (https://www.ieeg.org/). The NeuroVista ambulatory monitoring device consists of an implantable lead assembled in line with a telemetry unit and a personal advisory device. Data were acquired at 400 Hz using 16 channels (4 × 4 contact electrode strips) implanted bilaterally according to a standardized canine implantation protocol^14^. Preictal and interictal segments were extracted from the iEEG recordings of dogs with naturally occurring focal epilepsy. In line with previous investigations^11,15,16^ and the American Epilepsy Society seizure prediction challenge consensus, preictal segments consisted of recordings of 1 hour prior to seizure onset with a 5 min intervention time. Interictal segments were randomly chosen from the entire recording with a restriction of 4 hours before or after a seizure.

### Higher Order Spectra

Higher order spectral analysis is an advanced signal processing method that allows exploring the existence of quadratic (and cubic) non-linearities. In contrast to traditional power spectrum, which quantifies the power of a time series over frequency, HOS analysis employs the Fourier transform of higher order correlation functions investigating non-linear coupling information. The bispectrum splits the skewness (third order moment) of a signal over its frequencies, quantifying the coupling between a signal’s oscillatory components. The bispectrum, quantifying oscillatory relationships between basic frequencies *f*_1_, *f*_2_, and their harmonic component “*f*_1_ + *f*_2_”, is computed from the Fourier transform of the third-order correlation (1).

1 $$Bis({f}_{1},{f}_{2})=\mathop{\mathrm{lim}}\limits_{T\to \infty }(\frac{1}{T})E[X({f}_{1}+{f}_{2}){X}^{\ast }({f}_{1}){X}^{\ast }({f}_{2})]$$

where *X*(*f*) is the Fourier transform of a time series *x*(*t*), (*) is the complex conjugate, and *E* denotes the arithmetic average estimator.

### Higher Order Spectral Features

**Figure 1:**
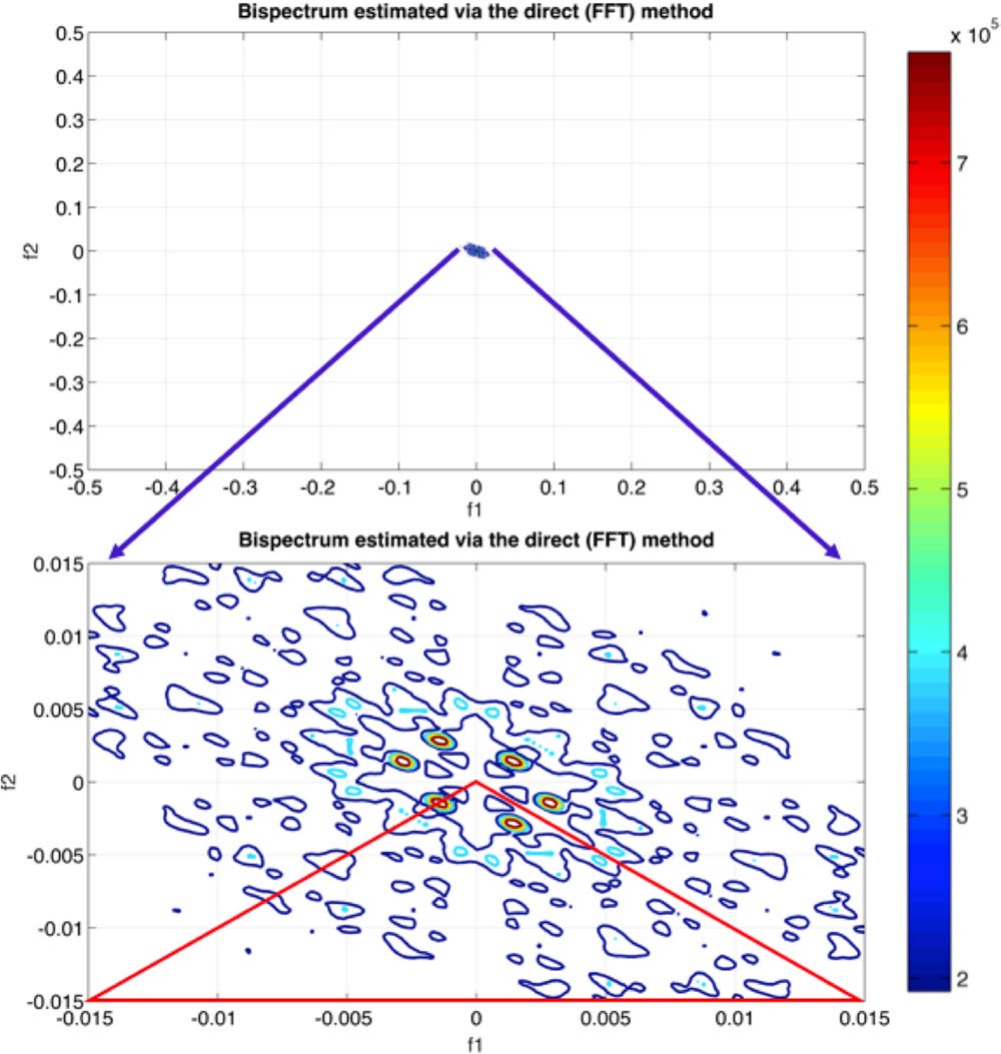
2D Bispectrum color map highlighting the non-redundant region used in feature extraction; FFT = Fast Fourier Transforms. Axes coordinates display relative/normalized frequencies where 0.5 represents the maximum frequency (180 Hz). Color indicates degree of coupling (Bispectral value) between *f*_*1*_ and *f*_*2*_.

In order to characterize and compare time series, quantitative features must be extracted from the bispectral density array. Bispectrum analysis yields a 2D mapping of the level of interaction between all frequency pairs in the signal. In order to characterize and compare time series, quantitative features must be extracted. In this work, three features were computed from the non-redundant region (shown in Fig. 1): the mean magnitude (*Mave*) of the bispectrum, the normalized bispectral entropy (*P*1) and the normalized squared bispectral entropy (*P*2). The mathematical equations of extracted features are briefly explained:

The first feature, bispectrum’s mean of magnitude (*Mave*) (2), has been used commonly to extract quantitative information from the bispectrum^13,17^.

2 $$Mave=\frac{1}{L}\sum _{{\rm{\Omega }}}\,|Bis({f}_{1},{f}_{2})|$$

where *L* = total number of sample points in the bispectral density array, and *Ω* refers to the non-redundant region defined in Fig. 1. In an attempt to extract regularity from bispectrum plots, normalized bispectral entropy (*P*1) and normalized bispectral squared entropy (*P*2) have been proposed recently^13^ and were used in this work:

3 $${P}_{1}=-\,\sum _{n}{p}_{n}log{p}_{n}$$

4 $${p}_{n}=\frac{|Bis({f}_{1},{f}_{2})|}{{\sum }_{{\rm{\Omega }}}|Bis({f}_{1},{f}_{2})|}$$

5 $${P}_{2}=-\,\sum _{n}\,{q}_{n}log{q}_{n}$$

6 $${q}_{i}=\frac{{|Bis({f}_{1},{f}_{2})|}^{2}}{{\sum }_{{\rm{\Omega }}}{|Bis({f}_{1},{f}_{2})|}^{2}}$$

where *n* = *0, 1*, … *n* − *1* and *n* is the number of bins.

A 30-sec non-overlapping moving window was used to compute the bispectrum and its subsequent features from epileptic canine iEEG recordings.

Zero-phase notch (cut-off frequency = 60 Hz) and band pass filtering in the frequency range [0.5–180] Hz were performed to keep signals’ phase intact. The higher order spectral analysis (HOSA) Matlab© toolbox was used to extract the bispectrum. Following equation (1), the bispectrum matrix was estimated for all possible frequency pairs (*f*_*1*_*, f*_*2*_) in the range [0.5–180] Hz based on the direct fast Fourier transform approach.

### Statistical analysis

To assess bispectrum related features’ capability in distinguishing between interictal and preictal iEEG recordings, a statistical analysis was performed to measure the level of statistically significant differences between the three features extracted from 30-sec non-overlapping windows.

Firstly, the general level of interaction between the type of recordings (interictal vs. preictal) and the values of each feature were evaluated for each dog, using one-way ANOVA. This analysis indicates whether there is a statistically significant difference between preictal and interictal recordings for each of the three features.

One-Way ANOVA was preferred over Student’s *t*-test, since the data were randomly and independently selected from the entire record and multiple features were compared.

Then, to assess the spatial localization of the change in bispectral features during the preictal period, the distribution of each feature for each hour of the preictal recordings (120 samples) was compared to the features for one hour of the interictal recordings, selected from the same electrode, with the restriction of 4 hours prior to the preictal time, by the Mann-Whitney U-Test. A *p*-value < 0.05 from this test indicates statistically significant difference.

Finally, for each feature, a color map of the brain was created to visualize the percentage of the seizures at each electrode for which the difference measured by the Mann-Whitney U-Test is significant at a confidence level of at least 95%. This representation allows a visualization and identification of the brain regions where the changes in bispectral features are most prominent during preictal periods.

### Seizure Prediction Algorithm

#### Network Architecture

**Figure 2:**
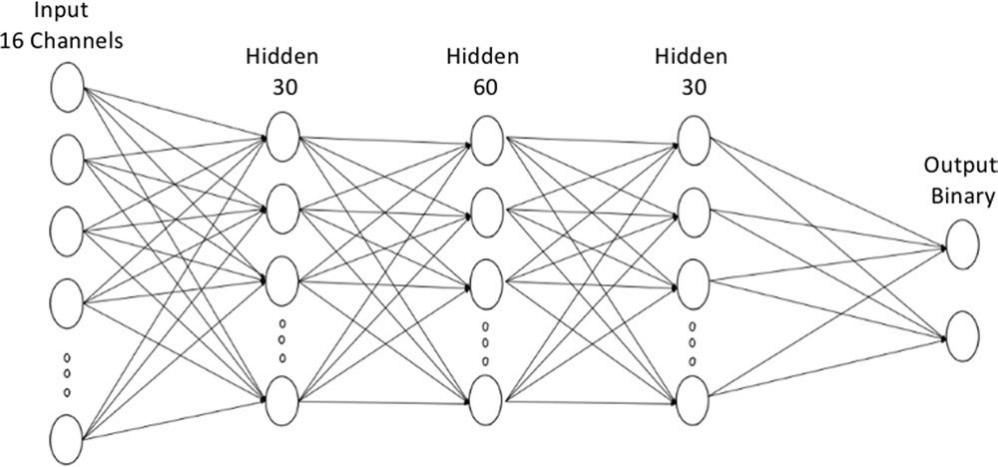
Artificial neural network architecture. The input layer consists of 16 nodes. The first, second, and third hidden layers respectively consists of 30, 60, and 30 nodes. The output layer features 2 nodes for a binary classification.

To assess the feasibility of seizure prediction based on bispectral features, a 5-layer MLP neural network classifier was trained to differentiate preictal and interictal recordings. Different classifier configurations were trained for each feature. The input layer consisted of 16 nodes (16 channels). The first, second and third hidden layers, respectively, consisted of 30, 60, and 30 nodes (ReLu activation function). The output layer contains 2 nodes for a binary decision function (Preictal vs Interictal). A stochastic gradient decent optimizer was used during backpropagation. The fitness function was the classification cross entropy. Training iterated through 10,000 epochs with a learning rate of 0.001 and a training and validation batch size of 200 samples. Figure 2 shows the architecture of the implemented neural network. All algorithmic development steps were performed on PyTorch, an open source Python-based machine learning library.

#### Data Splitting and training strategy

Held-out validation and test were performed. A total of 45 seizures were included in the analysis. A subject-specific algorithm was implemented. Features were extracted using a 30-sec non-overlapping moving window (total of 10’800 classification samples). Training, validation, and testing data were respectively in the following proportions: 40%, 30%, and 30%. To avoid any contamination, time correlation or leakage, the data (train, validation, and test) were split on a seizure per seizure basis.

More specifically, the whole preictal period (1 hour segmented using a 30-sec non-overlapping window) of a considered seizure was used for either training, validation, or testing. Splitting samples from the same preictal period (although not identical) into training, validation, and testing may prompt the classifier to learn temporal correlations rather than class information. This, in turn, would result in overoptimistic classification performances. The proposed strategy ensured that preictal samples originating from seizures used in training were neither assessed during validation nor testing.

## Results

### Statistical analysis

#### ANOVA – Global assessment of significance

**Figure 3:**
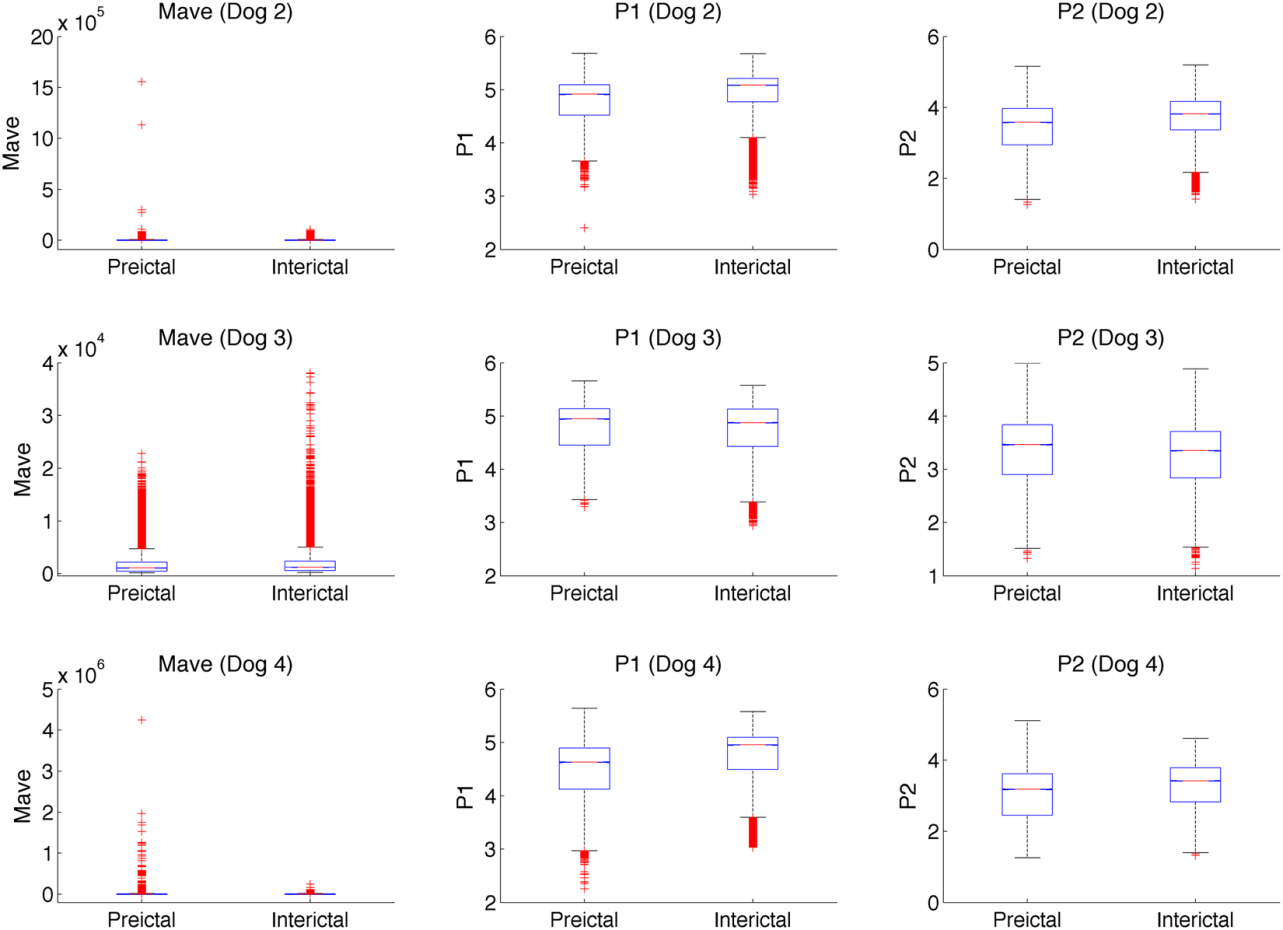
Box and Whisker plots for all features from all three dogs. The red central mark indicates the median, the bottom and top edges of the box indicate the 25th and 75th percentiles, respectively, and the whiskers extend to the most extreme data points to a maximum of 1 times the interquartile range. Outliers are points located beyond the whiskers and are marked with a red ‘+’. The columns from left to right show plots for *Mave*_,_ *P1* and *P2*, while the rows correspond to the 3 dogs. All available seizures are included in these box plots.

One-way ANOVA tests were conducted for each dog to compare HOS features extracted from preictal and interictal iEEG recordings. Results from these variance tests are shown in Table 1 and in the box-and-whisker plots in Fig. 3. For the bispectral magnitude (*Mave*), the distributions from two of the three dogs show a slight decrease in magnitude during the preictal phase, while the ANOVA tests for all three dogs indicate that preictal and interictal *Mave* distributions are statistically different at a confidence of at least 95% (Dog 2: *F*_*1,4078*_ = 6.07, *p* < 0.05; Dog 3: *F*_*1,4078*_ = 167, *p* < 0.001; Dog 4: *F*_*1,2638*_ = 18.5, *p* < 0.001). As for the normalized bispectral entropy (*P1*), distributions from all three dogs show a general decrease in *P1* values during the preictal phase and the ANOVA tests confirm that the difference in *P1* distributions between preictal and interictal recordings is statistically significant in all three dogs (Dog 2: *F*_*1,4078*_ = 2480, *p* < 0.001; Dog 3: *F*_*1,4078*_ = 98.3, *p* < 0.001; Dog 4: *F*_*1,2638*_ = 2340, *p* < 0.001). Finally, the normalized squared bispectral entropy (*P2*) values generally decrease while variances of the distributions increase during transition to seizure. The differences between the two *P2* distributions are statistically significant for all three dogs (Dog 2: *F*_*1,4078*_ = 2800, *p* < 0.001; Dog 3: *F*_*1,4078*_ = 346, *p* < 0.001; Dog 4: *F*_*1,2638*_ = 1360, *p* < 0.001). These strong significant differences imply that interictal and preictal recordings are statistically distinguishable based on the three HOS features tested.

**Table 1:** Mean values of HOS features and One-Way ANOVA global statistical analysis results. Mean values and standard deviation of HOS features for each dog were computed using recordings from all available seizures. Independent Analysis of Variance tests comparing preictal and interictal HOS feature distributions were conducted on recordings of each dog. Nb. Seizures: total number of seizures; the numbers in parentheses indicate the total number of 30-sec data samples used in the comparison; F: F statistic of one-way ANOVA; *p*: *p*-value of one-way ANOVA indicating probability that the null hypothesis (H_O_) is falsely rejected (H_O_: preictal and interictal feature distributions have equal means), *p-value is less than Matlab’s digit precision = 4.9407e–324.

Dog ID	Nb. Seizures	Mave			P1			P2		
		Pre	Inter	F, p	Pre	Inter	F, p	Pre	Inter	F, p
0002	17 (4,080)	3.31e3 ± 3.30e3	3.49e3 ± 2.57e3	F = 6.07, p = 0.0138	4.79 ± 0.30	4.94 ± 0.21	F = 2480, p = 0*	3.45 ± 0.53	3.71 ± 0.45	F = 2800, p = 0*
0003	17 (4,080)	1.79e3 ± 1.04e3	2.02e3 ± 1.24e3	F = 167, p = 4.113e-38	4.49 ± 0.24	4.75 ± 0.28	F = 98.3, p = 3.67822e-23	3.35 ± 0.46	3.26 ± 0.43	F = 346, p = 5.50e-77
0004	11 (2,640)	7.44e3 ± 1.46e3	5.99e3 ± 4.33e3	F = 18.5, p = 1.75e-5	4.05 ± 0.38	4.72 ± 0.34	F = 2340, p = 0*	3.04 ± 0.59	3.28 ± 0.51	F = 1360, p = 3.26e-292

#### Mann Whitney – Inter seizure assessment of significance

**Figure 4:**
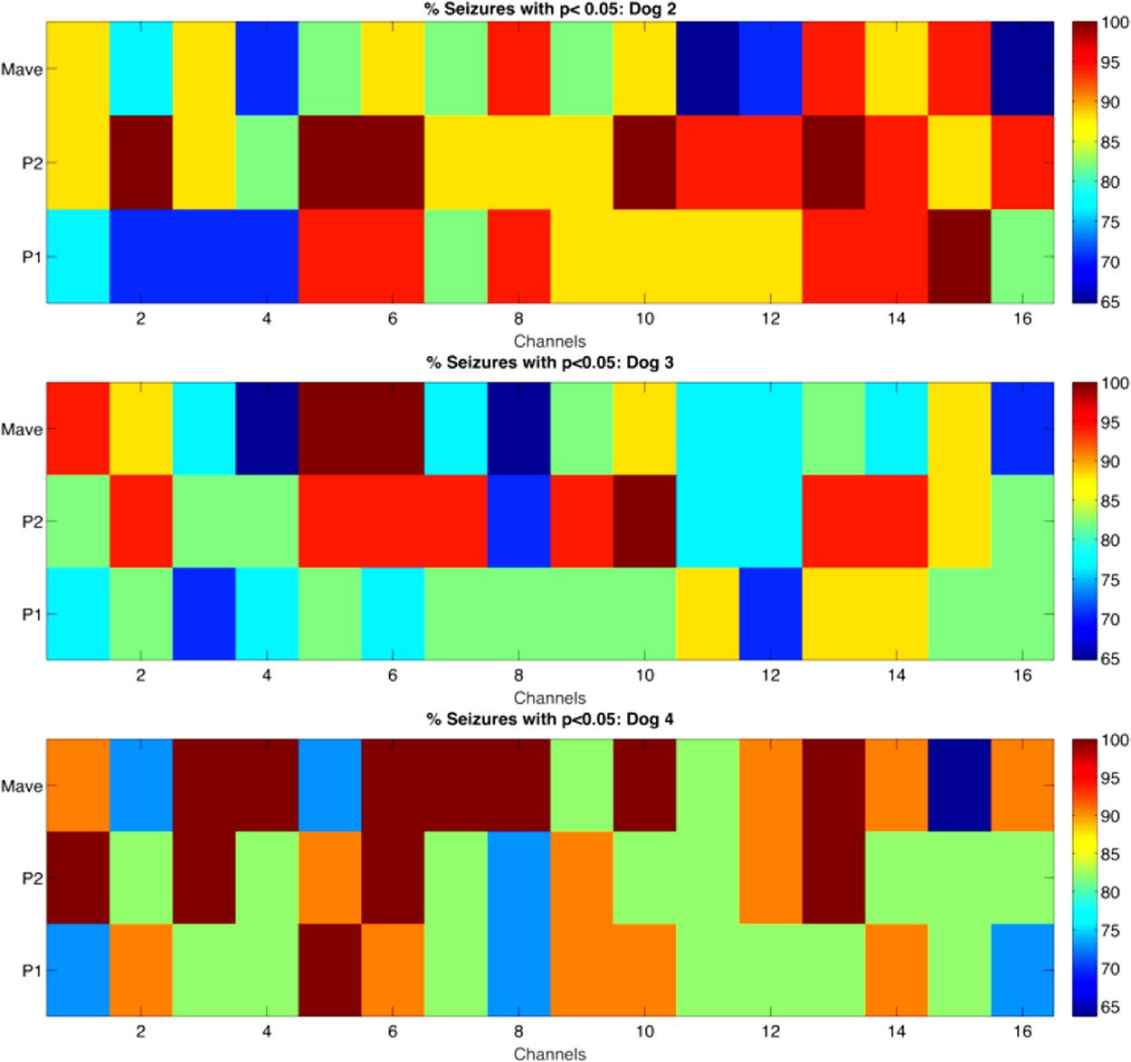
Mann-Whitney statistical test results: percentage of predictable seizures using each of the extracted features (p < 0.05). From top to bottom: Dog 2, Dog 3, Dog 4. Each cell represents a combination of a HOS feature and a contact. Dark red color indicates that 100% of seizures showed a statistically significant change in that feature during the preictal period at that specific contact.

The second statistical test aimed to evaluate the potential patient-specific seizure prediction capability of the three HOS parameters by analyzing the spatial distribution of the iEEG channels, for which the bispectral changes are most prominent. The HOS parameters extracted from 30-sec non-overlapping windows for a total of 45 preictal hours were compared to 45 interictal hours channel per channel. The Mann-Whitney U test was used to compare specific bispectral feature distributions for each seizure and each channel. The results of the specific statistical comparison tests are presented in Fig. 4. The colormaps represent the percent of predictable seizures, occurring in each dog, for which each specific feature distribution is statistically different (*p* < 0.05) during the preictal hour at that channel. For dog 2, the *P1* and P2 distributions change significantly during the preictal periods for 100% of the seizures (*n* = 17) at several contacts located in both hemispheres (Fig. 4, top).

Furthermore, these specific regions of consistent bispectral change coincide with the regions of most prominent cross-frequency phase-amplitude coupling (PAC) change, which we identified in an earlier study^11^. These regions include channels 2 and 5 in the left hemisphere and channels 10 and 14 in the right hemisphere. For dog 3, mean magnitude (*Mave*) and normalized bispectral entropy (*P1*) show significant distribution changes during preictal periods for 100% of the seizures (*n* = 17). As shown in Fig. 4 (middle), these distribution changes are most prominent at channels 1, 5 and 6 in the left hemisphere and channels 10 and 13 in the right hemisphere.

This spatial distribution of preictal bispectral feature change, again, coincides with the spatial distribution of preictal PAC change for this dog identified in our previous study^11^. Finally, for dog 4, the mean bispectral magnitude and normalized bispectral entropy showed most consistent seizure prediction potential. Once again, as shown in Fig. 4 (bottom), there was a statistically significant change in *Mave* and *P1* during progression to seizure for 100% of the seizures (n = 11) in bilateral regions, which coincide with those we identified as PAC change regions in Gagliano *et al*.^11^. These channels include 3, 4, 6, 7 and 8 in the left hemisphere and channel 13 in the right hemisphere.

### Seizure Prediction Algorithm

As previously mentioned, a 5-layer MLP was trained to classify interictal and preictal samples. As shown in Table 2, average test accuracies of 78.11%, 72.64%, and 73.26% were achieved using features *P1*, *P2*, and *Mave*, respectively. Table 2 reports performance results, in terms of accuracy, during training and testing, for all features from the 3 dogs. Training and testing performances were close in the case of *P1* and *P2*, whereas it was not in the case with the *Mave* feature, suggesting that the latter may be less useful for seizure prediction. An early stopping strategy was used during training. Training and validation were iterated through 10’000 epochs. Checkpointing was performed on a 10 epochs basis (save classifier model). The best model was chosen as the latest saved classifier model before validation loss starts increasing. The model was then assessed on held-out test data.

**Table 2:** Multilayer Perceptron-based classification results.

	P1		P2		Mave	
	Train Acc. (%)	Test Acc. (%)	Train Acc. (%)	Test Acc. (%)	Train Acc. (%)	Test Acc. (%)
Dog A002	84.23	76.71	79.81	77.61	94.23	61.84
Dog A003	71.71	67.23	75.58	61.52	79.26	67.15
Dog A004	90.89	90.40	83.36	78.78	94.58	90.80
Mean	82.8	**78.11**	79.58	**72.64**	89.36	**73.26**

## Discussion

In this work, we have examined the ability of HOS features in distinguishing preictal from interictal iEEG recordings in canines implanted with the NeuroVista ambulatory monitoring device. To our knowledge, this is the first investigation of the bispectrum within the context of seizure prediction. Unlike power spectrum (commonly used in forecasting studies), the bispectrum preserves phase information, which is useful for displaying quadratic nonlinear coupling between the different frequency components of the signal. Results highlight the feasibility of seizure forecasting, based on higher order spectra. These results compliment previous investigations of cross-frequency analysis, namely phase-amplitude coupling for seizure forecasting^10,11^. In addition, prominent performances of EEG-bispectrum features were reported within the context of EEG signal classification^13^. Chua *et al*. 2009 demonstrated a significant difference between EEG recordings from healthy and epileptic patients, using a one-way ANOVA test^13^.

**Figure 5:**
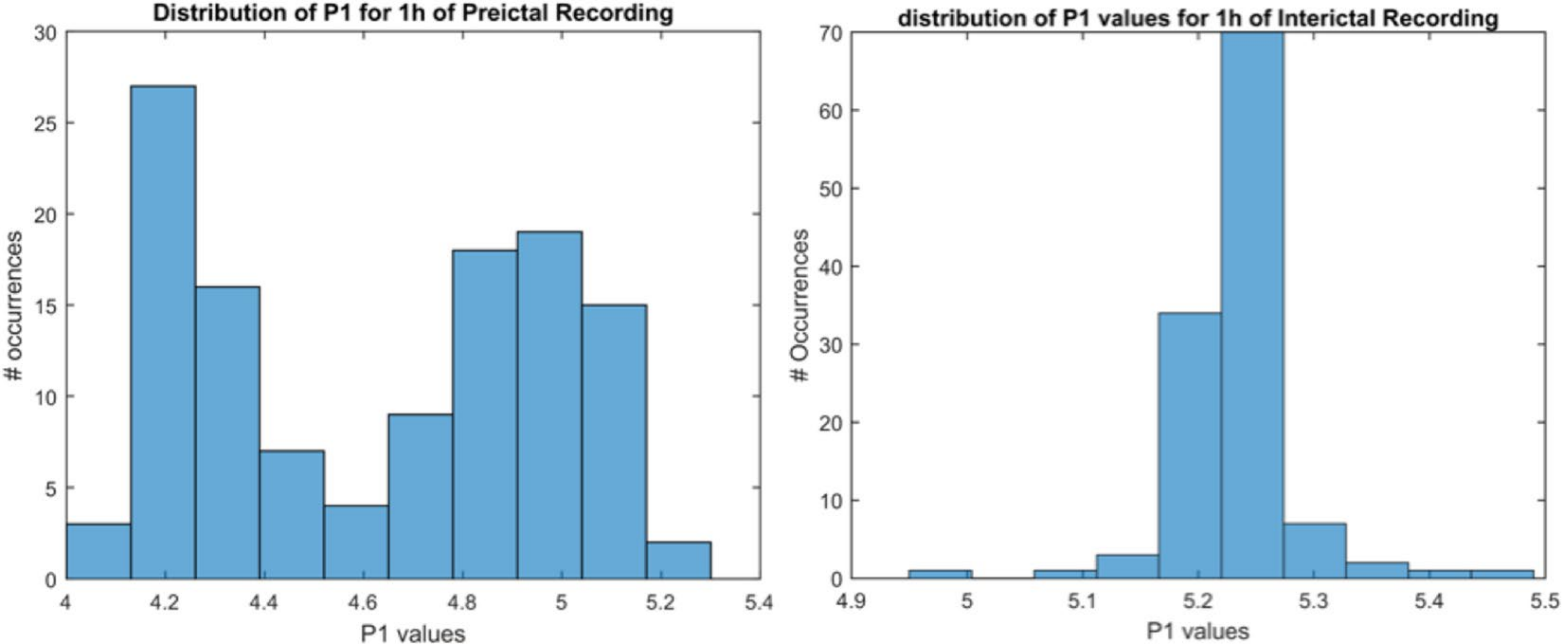
Distribution of *P1* values during preictal (left) and interictal (right) periods. Each distribution represents values extracted from 1 h of continuous recording from Dog 2.

ANOVA statistical analysis results revealed a general tendency for *P1* and *P2* features to decrease during the preictal state (decrease in mean amplitude for all 3 dogs). As these features display irregularity in the properties of iEEG signals, it seems that the iEEG characteristics tend to become more regular during the preictal state. These findings are in agreement with previous dimension analysis of EEG studies, which showed that seizures can be considered as emergent brain states with reduced complexity as compared to non-seizure activity^18,19^. As emphasized in^20^, it appears that a loss of complexity is associated with functional impairment of biological systems. In addition, Mann-Whitney test results confirmed the observed decrease of irregularity, while displaying a more normal distribution of interictal values as compared to preictal ones (Fig. 5).

The use of the NeuroVista database allowed investigating the bilateral nature of HOS changes. Although dogs were diagnosed with focal epilepsy^11,15^, bilateral preictal HOS changes were found in 2 of the 3 dogs. Interestingly, these findings correlate with our previous PAC-based preictal changes^11^. Unfortunately, we were unable to correlate these findings with respect to the exact location of the seizure onset zone, as this information is not provided within the dataset.

Recent reports have shown that high frequency oscillations can be used as a predecessor of seizure activity^21^. Considering the sampling frequency limitation imposed by the NeuroVista ambulatory monitoring device (Fs = 400 Hz), we were unable to explore quadratic non-linear coupling at the HFO level.

In this work, we did not explore the interaction among pre-defined frequency bands (standard iEEG frequency bands). The whole available frequency range was included in the analysis. The fact that standard iEEG frequency bands are used in power spectrum-based analysis does not necessarily justify their use in bispectrum analysis. Although this research avenue is tempting, it goes beyond the focus of this manuscript.

In this manuscript, we have demonstrated the suitability of MLP neural networks for the classification of interictal and preictal samples based on bispectrum-extracted features. Considering the image-based nature of bispectrum plots, it would be interesting to investigate the use of other types of neural networks’ architectures, namely, convolutional neural networks (CNNs). The design of seizure predictors, combining raw bispectrum plots and CNNs, is a tempting approach that may improve seizure prediction capabilities.

Each of the aforementioned features was used as an input to a seizure prediction algorithm. To avoid any bias, no previous assumption (based on the statistical analysis) was included during the seizure forecasting algorithm design. For each feature, all electrodes were used as inputs to a 5-layer MLP neural network. We ensured adequate performance evaluation and employed rigorous methodology to avoid reporting overoptimistic results: (1) data were split into training, validation, and testing; (2) Splitting was performed on a seizure per seizure basis to avoid leakage, or time correlation; and (3) Held-out validation and testing were performed. As in previous seizure forecasting investigations, results highlight that changes are not homogenous across the tested dogs and that subject-specific algorithms are required. It is worth mentioning that no post-processing has been performed in this work in an attempt to improve forecasting capabilities. Our objective was to test the capability of a neural network for individually classifying feature samples extracted from 30-sec iEEG samples as preictal or interictal. Future perspectives include extrapolating this methodology to a continuous seizure prediction framework which takes into account time-based modulation of HOS features. Such algorithms could be implemented into closed-loop intervention systems for advisory or intervention purposes.

## Conclusions

In conclusion, this work can be considered as a proof of principle study on the feasibility of seizure prediction based on HOS features. We have demonstrated statistically significant differences between preictal and interictal iEEG recordings for all the computed features. In addition, HOS analysis showed promising forecasting performances, when used as inputs to a neural network classifier. Additional studies assessing the performance of HOS features for seizure forecasting, ideally in a quasi-prospective setting, are necessary to advance the development of seizure advisory/intervention devices.
